# Social Support Network among People Living with HIV/AIDS in Iran

**DOI:** 10.1155/2013/715381

**Published:** 2013-04-28

**Authors:** Ameneh Setareh Forouzan, Zahra Jorjoran Shushtari, Homeira Sajjadi, Yahya Salimi, Masoumeh Dejman

**Affiliations:** ^1^Social Determinants of Health Research Centre, Department of Social Welfare Management, University of Social Welfare and Rehabilitation Sciences, Tehran, Iran; ^2^Department of Social Welfare Management, University of Social Welfare and Rehabilitation Sciences, Tehran, Iran; ^3^Social Determinants of Health Research Center, Department of Social Welfare Management, University of Social Welfare and Rehabilitation Sciences, Tehran, Iran; ^4^Department of Epidemiology & Biostatistics, Public Health School, Tehran University of Medical Science, Tehran, Iran; ^5^Department of Epidemiology & Biostatistics, Public Health School, Kermanshah University of Medical Science, Kermanshah, Iran; ^6^Social Determinants of Health Research Center, University of Social Welfare and Rehabilitation Sciences, Tehran, Iran

## Abstract

This study considers social network interactions as a potential source of support for individuals living with HIV/AIDS in Iran. This cross-sectional study was conducted on 224 people with HIV/AIDS who refer to behavioral counseling centers. Participants were randomly selected among all people with HIV/AIDS from these centers. Relatives were more reported as sources of support than nonrelatives. They were closer to participants, but there was difference between the closest type among relative and nonrelative supporters (*P* = 0.01). Mean of functional support with considering the attainable range 0–384 was low (126.74  (SD = 76.97)). Social support of participants has been found to be associated with CD4 cell count (*P* = 0.000), sex (*P* = 0.049), and network size (*P* = 0.000) after adjusted for other variables in the final model. Totally, in this study, many of participants had the static social support network that contained large proportions of family and relatives. The findings contribute to the evidence for promotion of knowledge about social support network and social support of people living with HIV/AIDS.

## 1. Introduction

AIDS is a disease that affects not only physical health but also mental and social conditions of patients because of the negative attitude of society, discrimination, and stigmatization [1–3] especially in the developing countries [4]. People living with HIV/AIDS (PLWHA) may experience social drift [5], changing pattern of sexual behaviors and self-image. They may lose employment, financial resources, and even family and friends as major sources of support [1, 3, 6]. Approximately 22727 cases of HIV/AIDS have been identified in Iran [7]. It is estimated that this number will increase to 106000 patients up to 2015 [8]. On the other hand, due to progress of antiretroviral therapy that increases life expectancy in patients [9], improvement of the quality of life in PLWHA is important [10, 11]. Previous studies suggested that social support can be an important factor for influences on well-being and quality of life [12, 13]. Researchers have been provided evidence that social support as an aspect of psychological adjustment [14] can improve physical and psychological health outcomes, increasing motivation for treatment [15], self-care behaviors [13], and also prevention of transmission of infection during HIV/AIDS disease [16–18]. Many PLWHA may facilitate living with HIV/AIDS by their social support networks (SSN).

SSN as a subset of a larger social network, consisting of some individuals who are linked to ego and roles such as emotional support, financial aid, guidance, and advice offered to him/her in a variety of situations [19, 20]. SSN is assumed to be stable in terms of size, composition, and resources of support, except in times of developmental transitions or nonnormative life changes, such as loss and diseases [21, 22]. Network studies have shown that family, friends, and relatives are important sources of social support that can provide the different types of support [1, 8, 23–26]. But the literature has shown that the size of social support networks for PLWHA was smaller than non-PLWHA [8, 16], and that this change can directly affect the amount of social support provided. Despite the growing body of literature on the concept of social support, there is little information about the structure and function of SSN and related factors among the PLWHA in Iran. The aim of the present study was to determine characteristics of SSN and association of these characteristics to social support among PLWHA. 

## 2. Methods

### 2.1. Study Site and Procedure

BCCs in Iran are the centers that provide free counseling, prevention, and treatment services for people with high-risk behaviors, HIV-positive and AIDS patient. Most people with HIV/AIDS are covered by these centers. Behavioral counseling centers of Tehran University of Medical Sciences were chosen as a study site. 

 This cross-sectional study was conducted from June to December of 2011. The 224 participants were randomly selected from all PLWHA referring to these centers. All of them have been receiveing antiretroviral therapy. Inclusion criteria were as follows: age over 15 years old, disclosing their HIV status at least to one person, and being aware of their infection/disease for at least the past six months. Because of self-administrated measure, illiterate people were excluded. Written informed consent was obtained from all participants at the time of the survey. This study was approved by the Research Ethics Board at the University Social Welfare and Rehabilitation of Tehran. 

### 2.2. Measures

Social support was measured by the Norbeck Social Support Questionnaire (NSSQ) which assesses supportive relation between the participants and the members of their social network. The questionnaire is composed of different types of social support: emotional support, instrumental support, functional support (emotional plus instrumental support), structural support (network size plus duration and frequency of contact), and loss support. The reliability and validity of the NSSQ were confirmed in other studies [27, 28]. Reliability and validity of the Farsi version of this questionnaire (NSSQ) have been evaluated and approved [29]. In the present study, Cronbach's alpha scores for subscales have ranged from 0.86 to 0.95.

The questions of structural subscale in NSSQ were used for measuring characteristics of SSN [3]. It is classified into questions about network size, network tie, network composition, duration of tie, and frequency of contact with network members. It must be mentioned that the Loss support subscale was eliminated because many participants had missing responses.

For network size, participants were asked to list people who they came in contact with in routine life and their relationship such as spouse, parent, and sibling (up to 24). 

Network composition was measured to determine which types of relations are more in the personal network of participants. It was categorized into two major groups: relative (as parent, spouse, child, sibling, and kin) and nonrelative (as friend, coworker, neighbor, advisor, doctor, and social organization). 

Network members are not necessarily a source of social support, unless the person perceives them as an available or suitable source of support for his/her needs. Network tie was used to refer to people in the personal network who have been providing support for participants. For instance, if a participant identified three supporters, three ties were produced for this participant. Then, it was asked that “how many of the people in your personal network provided support for you?”

 Frequency of contact was asked with questions like: “how often are you in contact with each of the listed people in your personal network?”. Four types of contact times (daily, weekly, monthly, and yearly) were specified. Type of contact was determined with three types of face-to-face, phone, and letter contact (by mail or e-mail).

 Tie duration refers to how long a tie among participants and their network members is there. Accordingly, the participants were asked about the duration of tie, which were categorized as “less than one year” (as short time), “one to five years” (as medium time), and “more than five years” (as long time).

Demographic and disease characteristics of the participant including sex, age, marital status (single, married), number of years of education, number of household members (without considering the participant), CD4 cell counts, and route of transmission of HIV (sexual relationship, inject drug user, and other (tattooing, mother to child, and dental services)) were also collected Also as a proxy of socioeconomic status, household level infrastructure was divided on the number of household members (considering participant) that it constructed area per capita of house. It was categorized into three groups: low (if the per capita was less than 10 meter), moderate (if the per capita was between 10 to 30 meter), and high (if the per capita was more than 30 meter) [30].

### 2.3. Data Analysis

Demographic characteristics and the size, composition, frequency of contact, and duration of tie of SSN were tabulated. Multiple linear regressions were also performed (considering the establishment of the required assumptions) to identify the association between demographic variables, characteristics of social network, and functional social support. Age, sex, number of household members, CD4 cell count, marital status, and number of years of education were used as independent variables. The following variables were entered into the model as the dummy variables: route of transmission of HIV and socioeconomic status. The baseline level for these variables was “sexual relationship,” “low,” respectively. Data were analyzed with using Statistical Package for the Social Sciences (SPSS) version 11.5. significant level was at 0.05.

## 3. Result

Response rate of 224 participants at the sampling was 96%. The mean of age of the participant was 35 (SD = 8.5) years, 68.3% were female and 31.7% male. Demographic characteristics of participants were presented in Table 1.

### 3.1. Network Size

Personal network size of participants was shown in Table 2. The mean network size was 8.1 (SD = 3.6). Nearly 88% reported four or more people in their personal network. Only two participants listed 24 people, and one reported only one member in his/her personal network. Totally, all participants named 1755 people as network members.

### 3.2. Network Composition

Majority of participants named relative (as parent, sibling, spouse, etc.) in their personal network (98.6%). Proportion of sibling was the most among relative composition (20.5%). Among nonrelatives, the proportion of friends was more than others in the personal network of PLWHA (20.2%). Composition of relation and sources of support (among participants and their network members) were shown in Table 3.

### 3.3. Network Ties

Table 3 presents the result of network tie. In this study, 1361 ties were identified by participants. In other words among all of the above mentioned network members (*N* = 1775) 1361 network members provided support for the participants. Overall proportion of the network tie was 77.5%. They are reported 72% relative and 28% nonrelative as a source of social support. It is apparent that relatives more frequently provided support than nonrelatives.

### 3.4. Frequency of Contact

The frequency of daily (45.1%) and face-to-face (69.4%) contact was greater than the other types of contact.

Mean face-to-face contact was 5.87 (SD = 3.37), and mean of phone contact was 2.44 (SD = 2.5).  Table 4 shows the results in detail.

### 3.5. Duration of Tie

 About duration of relationship, participants have known majority of their network members 78.9% for long time (more than five years), 15.9% for medium time (one to five year), and 5.2% for short time (less than one year). 

### 3.6. Social Support

Table 5 shows the scores of different types of social support in detail. The mean of functional support of participants considering the attainable range of this subscale (0–384) was low. Also, mean of structural support considering its' attainable range was low too. In term of sources of support, mean of functional family support was greater than friend support (74.48 (SD = 39.45) versus 22.82 (SD = 18.41)), and this finding was seen about structural support (49.38 (SD = 22.29) versus 11.86 (SD = 7.04)).

 In the regression analysis that included demographic characteristics as independent variables (Model 1, Table 6), marital status (*P* = 0.036), CD4 (*P* = 0.02), route of transmission (*P* = 0.048), and number of household members (*P* = 0.045) were significant predictors of social support. In Model 2, network size (*P* = 0.000) was a significant predictor of social support. The final model revealed that marital status and number of household members, which were significant predictors of social support in the first model, were not shown significant association when networks' characteristics as network size and frequency of contact were added in the model, but the network size (*P* = 0.000), CD4 (*P* = 0.000), and sex (*P* = 0.049) remained a significant predictors of social support (see Table 6). 

## 4. Discussion

This study has used personal network analysis to explore the characteristics of SSN of PLWHA. Our finding suggests that the mean of support network size for PLWHA was more than that reported in other studies [8, 27], but it was smaller than the mean of network members reported in a study of non-PLWHA in Iran [26]. These differences can be partly attributed to that, first, there was no limit of time as “in the last six months” for listing network members in the questionnaire and the number of people who could be listed in the personal network was more than what was used in the study of PLWHA in China (1–24 versus 1–13) [8].

According to the literature the size of one's social network seems to be less important to health than the network tie and its composition [31]. Hence, more emphasis in this study was to identify network ties. The findings showed that there are no isolated participants, who did not have any network member. Considering network ties as supporters, 3 participants named some people in the list of personal network but did not consider them as supporters, and 1 participant reported only one people as supporter. 

The proportion of relative was more than other sources of support. In other words, relative members were the most important people in the life of PLWHA. This is similar to the ranking of supporters in the study of PLWHA in China [8] and USA [32]. Because of the strong and stable nature of ties between family members, it is unlikely that available support and relations to relatives can be replaced by other network members [33]. Therefore, relative members have to understand the needs of HIV/AIDS patients and help them to manage the new conditions of life with HIV/AIDS in order to improve the psychological and social conditions [34]. These supports may help increase the adherence to treatment and finally prevention of transmission of the infection. Also, it must be noticed that Iranian society is characterized by different environmental, cultural, and political settings, and that these characteristics are reflected in nature of social relations. For example, in a study conducted by Bastani, the relative network plays a significant role as a system of support in the life of middle class people of Tehran city [26]. Iranian and Islamic cultures through the formal and informal religious teachings may influence the pattern of social relations especially in the disease condition.

The literature suggests that high frequency of contact among network members increases awareness of needs and resources and facilitates the exchange of aid [25]. But in the present study, the finding showed participants with lower CD4 had limited relations. It can be as a result of rise of clinical symptoms, change in body image, fear of stigma, and rejection and transmission of infection [35]. Also, people who had the lower years of education have more frequent face-to-face contact with their network members. This finding might be related to the composition of their networks. They were more report larger number of kin and neighbors in their networks which increase their chance for having more frequent contact. Likewise, participants had frequent face-to-face contact with close members as relative, advisor, and friend.

Some emphasized that the longevity of the relationship with the network members is an important dimension to the social support (e.g., I know him/her for a long time) [36]. In this study, participants had long duration of tie with the majority of their network members (78.9%) which is similar to the Australian study [36]. This may be the result of the presence of more relatives within their networks. Ties that have existed for a long time may have more impact on a person's behavior than ties that have existed for a short time. The duration of tie is significantly associated with the size of the network; that large network may contain more relative. It must be mentioned that many of these people due to depression and distress of their disease willing to have limited relations with new others that it may lead to statically social network with long duration of tie. 

Also, the results showed that mean of social support of PLWHA considering the range of functional support score was very low. It is similar to that reported in the literature [27]. In contrary with some literatures which suggested that the friend is an important source of support than family members in this study as other studies [8, 16, 32, 36–38], it was found that family members provided more support than friends for PLWHA. These differences may be due to dynamic and multidimensional nature of social support [3, 39, 40] that perceptions of social support change over time and are dependent on the cultural condition [8] of each society and individual circumstances [1]. 

Social support of PLWHA has been found to be associated with CD4 cell count, sex, and network size after being adjusted for other variables in the final model. In Model 1, however, there was a significant association between social support and some demographic variables (marital status, CD4, contamination, and number of household members) but they low explained the variability of social support (adjusted *R*-squared = 0.05). However, the proposed models in this study have not explained a large percentage of the variance of social support, but our findings suggest the significant variables in models as determinants of social support in PLWHA. A reason for the low values of adjusted *R* ^2^ could be the possible nonlinear association between the mentioned independent variables and social support. This issue also reveals the need to search for other social support predictors among PLWHA in future studies. 

In summary, many of the PLWHA in this study had static SSN that contained large proportions of family and relatives. However, nearly the majority of participants did not have strong SSN with readily available sources of support. These findings highlighted the importance of addressing related factor to social support, especially importance of having more individuals as family, friends, and advisors members in support networks for PLWHA. Our study suggested that several sources of support which they can turn to if needed, may increase their perception of social support. Also the lower levels of the social support score of PLWHA in this study demands the relevant attention to these vulnerable people.

The following limitations apply to the findings of this study. First, this study was a cross-sectional study, so causal links as well as true mediation between the variables could not be determined between characteristics' demographics, social network, and social support. In addition, this study did not address other possible factors that might have been related to social support and could explain more variance in the outcome. Further, because this study targeted PLWHA, who are referring to BCC of Tehran University of Medical Sciences, the findings cannot be generalized to other PLWHA. Other limitations are self-report measures. Despite these limitations, this study is one of only a few that has explored social support and its related factors among PLWHA who live in Iran. The findings of this study contribute to the evidence for promotion of knowledge about SSN of PLWHA. 

## Figures and Tables

**Table 1:** Demographic characteristics of participants in study.

Variable	*N*	Mean (SD) or %
Age	215	35 (8.5)
Number of household members	214	3.64 (1.82)
Number of years of education	212	9.8 (3.75)
CD4 cell count	215	306.7 (252.9)
Network size	215	8.1 (3.6)
Route of transmission		
Sexual relationship	95	44.2%
Inject drug user	84	39.1%
Other (tattooing, mother to child, and dental services)	36	16.7%
Marital status		
Married	101	47%
Single	114	53%
Sex		
Female	72	33.5%
Male	143	66.5%
Socioeconomic status		
Low	17	7.9%
Moderate	140	65.1 %
High	58	27%

**Table 2:** Network size of participants in study.

Network size	Frequency	Proportion (%)	Cumulative proportion (%)
1	1	.5	.5
2	2	0.9	1.4
3	10	4.7	6.
4	14	6.5	12.6
5	22	10.2	22.8
6	19	8.8	31.6
7	32	14.9	46.5
8	29	13.5	60
9	30	14.0	74
10	18	8.4	82
11	6	2.8	85.1
12	9	4.2	89.3
13	7	3.3	92.6
14	4	1.9	94.4
15	5	2.3	96.7
16	2	.9	97.7
17	1	.5	98.1
18	1	.5	98.6
23	1	.5	99.1
24	2	.9	100.0

Total	215	100.0	

**Table 3:** Network composition and tie of participants in study.

	Network composition		Network tie	
	*N *	Proportion %	*N *	Proportion %
Relative	212	98.6	980	72
Parent	169	78.6	254	18.6
Spouse	104	48.4	104	7.6
Child	57	26.5	108	7.9
Sibling	188	87.4	280	20.5
Kin	104	48.4	234	17.1
Nonrelative	171	79.5	381	28
Friend	126	58.6	275	20.2
Coworker	38	17.7	77	5.6
Advisor	10	4.7	14	1
Neighbor	9	4.2	10	0.73
Social organization staff	0	0.00	0	0.00
Doctor	4	1.9	5	0.36

**Table 4:** Frequency of contact of participants in study.

Time of contact			Type of contact		
	*N *	Proportion %		*N *	Proportion %
Daily	669	49.1	Face-to-face	934	68.6
Weekly	323	23.7	Phone	427	31.3
Monthly	204	15	Mail and e-mail	0	0.0
Yearly	165	12.1			

Total	1361	100		1361	100

**Table 5:** Perceived social support of participants in study.

Social support	Mean (SD)	Min–max	Attainable range
Functional support	126.74 (76.97)	0–549	0–576
Emotional support	84.02 (52.65)	0–377	0–384
Instrumental support	42.72 (26.29)	0–172	0–192
Structural support	77.47 (39.98)	8–229	0–240

**Table 6:** Multiple linear regressions for characteristics of social support network and social support.

Dependent variable	Social support β*		
	Model 1	Model 2	Model 3
Adjusted R2	0.05	0.41	0.46
Age	−0.008	—	0.067**
Sex			
Female	—	—	—
Male	−0.041	—	−0.095**
Marital status			
Single	—	—	—
Married	0.146**	—	0.061
Number of years of education	−0.043	—	0.031
Number of household members	0.143**	—	−0.019
CD4 cell count	0.160***	—	0.221***
Route of transmission			
Sexual relationship	—	—	—
Injection	−0.145	—	−0.016
Other	−0.133**	—	−0.002
Socioeconomic status			
Low	—	—	—
Moderate	0.002	—	−0.0104
High	−0.124	—	−0.116
Network size	—	0.661***	0.659***
Frequency of contact	—	−0.022	−0.023

*Regression coefficients (*β*) in relation to functional social support.

**P value < 0.05.

***P value < 0.01.
